# Editorial – 20 years Journal of Integrative Bioinformatics

**DOI:** 10.1515/jib-2025-0034

**Published:** 2025-07-09

**Authors:** Ralf Hofestädt

**Affiliations:** University of Bielefeld, Bielefeld, Germany

Based on the human genome project, *Bioinformatics* was established as a new scientific area during the ‘90s of the last century. In 1993 the *German Society of Computer Science* (GI) founded the new section *Informatik in den Biowissenschaften* (Computer Science in the Biosciences, GI FG 4.0.2). Based on these activities the *German Conference on Bioinformatics* (GCB) was founded 1996 in Leipzig. The GCB represents the annual Bioinformatics meeting in Germany until now. During this time the internet emerged, which allowed the easy access to relevant molecular databases, information systems and analysis tools. Based on this electronic infrastructure new methods and concepts for the user-specific information fusion process had been and have to be developed.

Based on these activities we organised a Dagstuhl seminar in 2004 (http://www.dagstuhl.de/04281/). The seminar was called *Integrative Bioinformatics* and organised by Prof. Nikolay Kolchanov and Prof. Ralf Hofestädt. We invited international scientists and discussed the current and future aspects of molecular information fusion. The key result of this meeting was the decision to come up with a new online journal to focus on this new topic of Integrative Bioinformatics. Furthermore we decided to establish an annual *International Symposium on Integrative Bioinformatics*. During this time the infrastructure for online journals was not available and we implemented our own software tool. The first issue of the *Journal of Integrative Bioinformatics* (JIB) was published in 2004 and presented invited talks of participants of this Dagstuhl seminar (https://www.degruyter.com/journal/key/jib/1/1/html). A few years later, we received the PubMed registration for our self-made JIB journal.

Over the years, the situation for scientists has changed as the Journal Impact Factor (JIF) has become increasingly important. This was one reason to shift our journal to the publishing house of De Gruyter. Thanks to this cooperation, we were able to obtain a impact factor for our journal.

With the current issue, we are pleased to announce the 20-year history of our work. The selected papers of this issue try to show the evolution of the key idea of information fusion in this area of research. In the beginning, federated database systems were discussed as a solution of information fusion. But a lot of disadvantages showed up – data security is one of these disadvantages. The answer was the implementation of Bio Data Warehouses. The paper of Schueler et al. is showing how an agile, participatory information fusion process enabled IPK to build a sustainable, FAIR-compliant research data infrastructure balancing operational needs and innovation. Looking to the papers which were published in JIB over the years we can see that most of the papers integrate the relevant data and tools by user specific workflows. The paper of Shilenok et al. is a nice paper which represents this kind of activities. Another highlight of our discussion of 20 years JIB is the system VANTED and the discussion about sustainable software development by Schreiber et al. The VANTED system is one of the few systems still in use after all this time based on the idea to integrate metabolic pathways based on KEGG pathway data. Finally, we chose the paper of Hansel-Fröse et al. for this special issue to show that the Petri net approach is a powerful method for the analysis of biological processes.

After 20 years of work I would like to thank all of you for your help and work to build up and support the JIB journal. The future of biotechnology, molecular biology, and molecular medicine is based on a powerful electronic infrastructure. The JIB journal will continue to support these important activities.

